# Meeting at St. Bartholomew's Hospital

**Published:** 1913-01-04

**Authors:** 


### THE BRITISH HOSPITALS ASSOCIATION.

### Meeting at St. Bartholomew's Hospital.

A meeting of the Council of the British Hospitals Association was held at St. Bartholomew's Hospital on Saturday, December 28, 1912.

The Chairman of the Council, Dr. D. J. Mackintosh, Superintendent of the Western Infirmary, Glasgow, presided, and there were present representatives of voluntary hospitals both from London, the provinces, and Scotland. Several letters were read from absent members expressing regret at their absence and offering various suggestions.

Mr. Conrad Thies, the Honorary Secretary, presented a memorandum in reference to the returns which had been received from a large number of voluntary hospitals throughout the United Kingdom, which showed that the numbers of insured persons receiving treatment at the present time in these institutions were as follows :—In-patients, from 24 to 60 per cent., averaging 49 per cent.; out-patients, from 18 to 72 per cent., averaging 47 per cent.

The Council then proceeded to consider the question of the treatment of insured persons in the voluntary hospitals, and the Chairman made the following statement :—

"We are met to-day as a Council to consider what steps should be taken by the managers of the voluntary hospitals throughout the country with regard to the treatment of insured persons after January 15 next. I am sure the gentlemen present, who represent voluntary hospitals of various sizes all over the country, will take a broad view of the present situation, and will not suggest any procedure which would imperil the voluntary principle.

"The Chancellor of the Exchequer has recently stated quite definitely that the provision made by the National Insurance Act is for domiciliary treatment alone, and does not touch the important work which hospitals have been carrying on in the past, and which, it is hoped, they will continue to carry on in the future.

"No doubt a great deal will depend as to what action medical practitioners are going to take with regard to the working of the Act, but, whether they go on a panel or come to some other arrangement, the work of hospitals, so far as in-patients are concerned, will not be lessened either in amount or importance.

"I think this is a great opportunity which should not be lost of bringing to the notice of the public the inestimable value and need of the voluntary hospitals in this country; and, if it is made perfectly plain that no provision whatever has been made for institutional treatment under the Act, I think it would be premature for the managers of voluntary hospitals to interfere in any way at the present stage with the treatment as in-patients of insured persons who suffer from ailments which cannot possibly be treated efficiently in their own homes.

"We have to remember that about 50 per cent. of the patients in all our voluntary hospitals are not insured persons. If the Act is properly worked, hospitals should be relieved of the treatment of insured persons who are suffering from affections which can be equally well attended to at home by their own doctors, so that in that direction the Act will be a distinct boon to the hospitals; for the time and attention of the medical staff will be devoted entirely to the particular class of work for which such hospitals exist—namely, the treatment of serious accidents, acute diseases, and major surgical operations which cannot be attended to at their homes and that require skilled nursing."

After a full discussion it was moved by the Chairman, and resolved to make the following recommendations to the voluntary hospitals :—

(1) With reference to out-patients the Council is strongly of opinion that upon medical benefit under the National Insurance Act coming into force, insured persons should be examined by a medical officer, but except for accidents, emergencies, or such special treatment as can only be given in a hospital, they should no longer be received in the out-patient and casualty departments, unless accompanied by a certificate or introduced personally by the medical practitioner who is in attendance. In such cases after consultation they should be referred back to their medical practitioner, with an expression of the opinion of the hospital physician or surgeon on the case. And a list of all such insured persons and the practitioners by whom they are sent should be forwarded to the Insurance Committee of the district periodically.

(2) With reference to in-patients, insured persons, whose cases are urgent and in need of hospital treatment not provided for under the National Insurance Act, should be admitted as heretofore, and the hospitals should keep accurate records of all such persons admitted, and, if possible, the Approved Society to which they belong.

The Rules of the Association as revised by the Executive Committee were approved and passed by the Council.

It was decided to accept with thanks the invitation from the Rev. G. B. Cronshaw, Chairman of the Radcliffe Infirmary, to hold the next annual Conference at Oxford in June or July next, the arrangements being left to the Executive Committee.

The proceedings closed with a vote of thanks to the Governors of St. Bartholomew's Hospital for granting the use of a room for the Council meeting.

---

We are requested to correct a report which was published in some of the papers to the effect that the regulations recently formulated by St. Bartholomew's Hospital in reference to the treatment of insured persons after the 15th inst. were discussed by the Council.

The Council discussed the report which had been prepared by the Executive Committee, and they decided that as the circumstances of the various hospitals differ considerably, the regulations which they may adopt must be framed to meet the special requirements of each institution. They have accordingly confined their recommendations to the main principles which they think should govern the arrangements to be made, at least for the present. The Council will not fail to watch carefully the effects of the working of the Act upon the hospitals, and will again communicate their views whenever the circumstances render it necessary.

